# A clade uniting the green algae *Mesostigma viride *and *Chlorokybus atmophyticus *represents the deepest branch of the Streptophyta in chloroplast genome-based phylogenies

**DOI:** 10.1186/1741-7007-5-2

**Published:** 2007-01-12

**Authors:** Claude Lemieux, Christian Otis, Monique Turmel

**Affiliations:** 1 Département de biochimie et de microbiologie, Université Laval, Québec, QC, G1K 7P4, Canada

## Abstract

**Background:**

The Viridiplantae comprise two major phyla: the Streptophyta, containing the charophycean green algae and all land plants, and the Chlorophyta, containing the remaining green algae. Despite recent progress in unravelling phylogenetic relationships among major green plant lineages, problematic nodes still remain in the green tree of life. One of the major issues concerns the scaly biflagellate *Mesostigma viride*, which is either regarded as representing the earliest divergence of the Streptophyta or a separate lineage that diverged before the Chlorophyta and Streptophyta. Phylogenies based on chloroplast and mitochondrial genomes support the latter view. Because some green plant lineages are not represented in these phylogenies, sparse taxon sampling has been suspected to yield misleading topologies. Here, we describe the complete chloroplast DNA (cpDNA) sequence of the early-diverging charophycean alga *Chlorokybus atmophyticus *and present chloroplast genome-based phylogenies with an expanded taxon sampling.

**Results:**

The 152,254 bp *Chlorokybus *cpDNA closely resembles its *Mesostigma *homologue at the gene content and gene order levels. Using various methods of phylogenetic inference, we analyzed amino acid and nucleotide data sets that were derived from 45 protein-coding genes common to the cpDNAs of 37 green algal/land plant taxa and eight non-green algae. Unexpectedly, all best trees recovered a robust clade uniting *Chlorokybus *and *Mesostigma*. In protein trees, this clade was sister to all streptophytes and chlorophytes and this placement received moderate support. In contrast, gene trees provided unequivocal support to the notion that the *Mesostigma *+ *Chlorokybus *clade represents the earliest-diverging branch of the Streptophyta. Independent analyses of structural data (gene content and/or gene order) and of subsets of amino acid data progressively enriched in slow-evolving sites led us to conclude that the latter topology reflects the true organismal relationships.

**Conclusion:**

In disclosing a sister relationship between the Mesostigmatales and Chlorokybales, our study resolves the long-standing debate about the nature of the unicellular flagellated ancestors of land plants and alters significantly our concepts regarding the evolution of streptophyte algae. Moreover, in predicting a richer chloroplast gene repertoire than previously inferred for the common ancestor of all streptophytes, our study has contributed to a better understanding of chloroplast genome evolution in the Viridiplantae.

## Background

Analyses of morphological and ultrastructural characters, and also of the information carried by gene sequences have established that green algae belonging to the class Charophyceae gave rise to the more than 500,000 land plant species currently inhabiting our planet [1,2]. Charophycean green algae and land plants form the green plant lineage Streptophyta [3], whereas most, if not all, of the other extant green algae belong to the sister lineage Chlorophyta [2]. In contrast to the large diversity of land plants, only a few thousands charophycean species are living today. Six monophyletic groups are currently recognized in the Charophyceae: the Mesostigmatales [4] represented by *Mesostigma viride*, a scaly biflagellate that has long been thought to be a member of the Prasinophyceae (the earliest-diverging lineage of the Chlorophyta) [5]; the Chlorokybales represented as well by a single species (*Chlorokybus atmophyticus*); the Klebsormidiales (3 genera, 45 spp.); the Zygnematales (~ 50 genera, ~ 6,000 spp.); the Coleochaetales (3 genera, 20 spp.); and the Charales (6 genera, 81 spp.) [6].

Recent phylogenetic studies of nuclear and organelle gene sequences have yielded conflicting results regarding the branching order of charophycean lineages and the identity of the charophycean lineage(s) that is/are sister to land plants. A phylogeny based on four genes from three cellular compartments (the nuclear 18S rRNA gene, the chloroplast *atpB *and *rbcL *and the mitochondrial *nad5*) supports the notions that the Charales are sister to land plants and that charophycean green algae evolved progressively toward a more elaborated cellular complexity, occurring sequentially as biflagellated unicells, cubical packets of two, four or eight non-flagellated cells (sarcinoid morphology), unbranched/branched filaments and complex branched thalli with parenchymatous tissue [4,7]. In this four-gene tree, inferred using the glaucocystophyte *Cyanophora paradoxa *and chlorophyte green algae as outgroup, the deepest branch is occupied by the Mesostigmatales, the Chlorokybales emerge just after the Mesostigmatales, the Zygnematales are resolved as the next divergence, and finally the Coleochaetales are sister to the clade uniting the Charales and land plants. Although the latter clade received strong support (> 90% bootstrap value), moderate bootstrap support was observed for the positions of the Coleochaetales, Zygnematales and Klebsormidiales. In contrast, our phylogenetic analyses of more than 50 genes and proteins derived from complete charophycean chloroplast genome sequences using *Mesostigma *as an outgroup do not indicate the existence of a sister relationship between the Charales and land plants [8,9]. These analyses, which are independently supported by structural genomic features, rather identified the Charales as a basal divergence relative to both the Coleochaetales, Zygnematales and land plants. The position of the Mesostigmatales in the Viridiplantae is also a matter of controversy. In the four-gene tree [4] and in trees based on 18S rDNA [10], actin genes [11] and concatenated chloroplast genes [12], *Mesostigma *represents the earliest divergence of the Streptophyta; however, separate phylogenetic analyses of multiple mitochondrial and chloroplast genes place theMesostigmatales before the split of the Streptophyta and Chlorophyta [13-17]. More recently, the finding that *Mesostigma *shares more ESTs with land plants than with the chlorophyte *Chlamydomonas reinhardtii *[18] as well as the discoveries of a multigene family (*BIP2*-like sequences) [19] and a *GapA/B *gene duplication [18,20] restricted to *Mesostigma *and streptophytes were interpreted as compelling evidence for the affiliation of this unicellular biflagellate with the Streptophyta.

We have undertaken the sequencing of the chloroplast genome from representatives of all charophycean lineages to unravel the phylogenetic relationships among these lineages and to gain insight into the origin of the highly conservative pattern displayed by land plant chloroplast DNAs (cpDNAs). We have reported thus far the cpDNA sequences of *Mesostigma viride *(Mesostigmatales) [13], *Chaetosphaeridium globosum *(Coleochaetales) [21], *Staurastrum punctulatum *and *Zygnema circumcarinatum *(Zygnematales) [22], and *Chara vulgaris *[8]. Comparative analyses of *Mesostigma *cpDNA (137 genes, no intron) with its land plant counterparts (110–120 genes, about 20 introns) revealed substantial changes in genome architecture (namely gene losses, intron insertions, and scrambling in gene order) [13]. *Chaetosphaeridium *and *Chara *cpDNAs more closely resemble their land plant counterparts than *Mesostigma *cpDNA at the levels of gene content (125 and 127 genes, respectively), intron content (18 introns in both cpDNAs), and gene order [8,21]. Like most land plant and green algal cpDNAs, *Mesostigma*, *Chaetosphaeridium*, and *Chara *cpDNAs exhibit a quadripartite structure that is characterized by the presence of two copies of a rRNA-containing inverted repeat (IR) separated by large (LSC) and small (SSC) single-copy regions. In contrast, the chloroplast genomes of the zygnematalean algae *Staurastrum *and *Zygnema *lack an IR [22]. Although their gene content (121 and 125 genes in *Staurastrum *and *Zygnema*, respectively) is similar to that found in *Chaetosphaeridium *and bryophyte cpDNAs, they feature substantial differences in overall gene order and intron content (8 and 13 introns). Comparative analyses of the abovementioned genomes revealed that the chloroplast genome of land plants inherited a myriad of characters from charophycean green algae [8,9].

In the present study, we describe the complete cpDNA sequence of *Chlorokybus atmophyticus *(Chlorokybales) and present chloroplast phylogenies based on the genomic data currently available for land plants, green algae, and other algae with primarily- or secondarily-acquired chloroplasts. We show that the *Chlorokybus *chloroplast genome bears close resemblance to its *Mesostigma *homologue and that the Mesostigmatales and Chlorokybales form a strongly supported clade that represents the deepest branch of the Streptophyta.

## Results

### Structural genomic features

**Figure 1:**
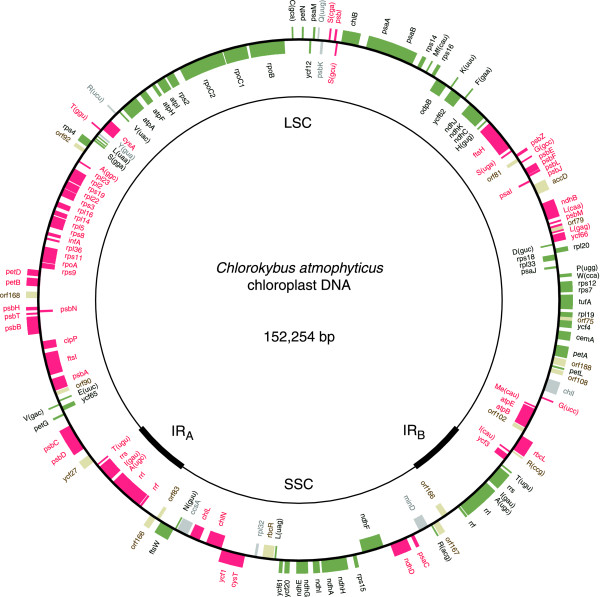
**Gene map of *Chlorokybus *cpDNA**. Genes (closed boxes) shown on the outside of the map are transcribed in a clockwise direction, whereas those on the inside of the map are transcribed counterclockwise. All ORFs larger than 75 codons as well as genes absent from *Mesostigma *cpDNA are represented in beige. Blocks of gene sequences colinear with *Mesostigma *cpDNA are shown as alternating series of green and red boxes. Genes present in *Mesostigma *cpDNA but located outside these conserved blocks are shown in grey. tRNA genes are indicated by the one-letter amino acid code (Me, elongator methionine; Mf, initiator methionine) followed by the anticodon in parentheses. The intron in *trnL*(uaa) is represented by an open box.

The *Chlorokybus *cpDNA sequence maps as a circular molecule of 152,254 bp with an overall A+T content of 63.8% (Figure 1). While this size is in the range expected for a streptophyte or chlorophyte genome, the nucleotide composition deviates slightly from the range (67.5–73.8% A+T) previously reported for streptophyte algae [8] and is most similar to the A+T content found for the ulvophyte *Pseudendoclonium akinetum *(62.3%) and the chlorophycean alga *Scenedesmus obliquus *(67.2%) [23]. Compared to its *Mesostigma *homologue, the *Chlorokybus *genome has a surplus of 33,894 bp and a deficit of 6.1% in A+T content. Both genomes are gene-rich and display the typical quadripartite structure found in streptophyte cpDNAs. In *Chlorokybus*, the two identical IR sequences of 7,640 bp are separated by a LSC region of 109,098 bp and by a SSC region of 27,876 bp. A total of 138 genes (not counting duplicate copies and unique ORFs) are encoded by *Chlorokybus *cpDNA: six reside in the IR sequence, whereas 23 and 109 are located in the SSC and LSC regions, respectively. The coding sequences of the 138 genes represent 58.8% of the genome size. Although genes are more tightly packed in the genomes of *Mesostigma *(73.2%), *Chaetosphaeridium *(76.9%), the liverwort *Marchantia polymorpha *(80.7%) and the chlorophytes *Nephroselmis olivacea *(68.7%) and *Scenedesmus *(67.2%), a similar level of compactness is observed for the other completely sequenced chlorophyte genomes (50.1–62.3%) [23]. At 435 bp, the average size of the intergenic spacers in *Chlorokybus *cpDNA is twice that found in *Mesostigma *cpDNA (221 bp). The intergenic regions account for most of the difference in nucleotide composition between the two genomes, with a variation of 13.5% in A+T content found for these regions (67.7% in *Chlorokybus *and 81.2% in *Mesostigma*) relative to only 4.6% for the coding regions (61.1% in *Chlorokybus *and 65.7% in *Mesostigma*). Like its homologues in *Mesostigma *and the prasinophyte *Nephroselmis*, the *Chlorokybus *genome is poor in introns; it carries a single intron, a group I intron in the *trnL*(uaa) gene. Homologous introns at identical position in this chloroplast gene have been reported in virtually all of the streptophytes studied thus far [8] and in a number of chlorophytes [23].

The gene repertoire of *Chlorokybus *cpDNA bears most similarity with that of *Mesostigma *cpDNA and features two genes (*rbcR *and *ycf27*) that have not been identified in the green algal and land plant chloroplasts investigated to date. These genes, which encode transcriptional regulators of the LysR and OmpR families, are present in the chloroplast genome of the glaucocystophyte *Cyanophora *[24], in all four completely sequenced red algal cpDNAs (*Porphyra purpurea *[25], *Cyanidioschyzon merolae *[26], *Cyanidium caldarium *[27] and *Gracilaria tenuistipitata *[28]), and in algal chloroplasts that were acquired by secondary endosymbiosis from the red algal lineage (the heterokont *Odontella sinensis *[29], the cryptophyte *Guillardia theta *[30] and the haptophyte *Emiliania huxleyi *[31]). Besides *rbcR *and *ycf27*, only two genes in *Chlorokybus *cpDNA [*accD *and *trnR*(ccg)] are missing from *Mesostigma *cpDNA; conversely, only three of the *Mesostigma *genes (*bioY*, *ssrA *and *ycf81*) are missing from *Chlorokybus *cpDNA. Together, *Chlorokybus *and *Mesostigma *cpDNAs encode seven genes [*bioY*, *rbcR*, *ssrA*, *ycf27*, *ycf61*, *ycf65 *and *trnA*(ggc)] that are absent from all other completely sequenced chloroplast genomes of green algae but are found in *Cyanophora*, red algal cpDNAs and/or the secondary chloroplasts derived from the red algal lineage.

At the level of gene organization, *Chlorokybus *cpDNA also most closely resembles its *Mesostigma *homologue (Table 1). In these two genomes, the IR and the corresponding single-copy regions display essentially the same gene content but vary in the order of 15 blocks of colinear sequences that collectively encode about 90% of the shared genes (Figure 1). Using GRIMM, a program allowing pairwise comparisons of gene orders, we estimated that a total of 14 inversions (4 in the SSC region and 10 in the LSC region) would be required to interconvert the chloroplast gene orders of *Chlorokybus *and *Mesostigma*. The *Chlorokybus *genome is more rearranged than is its *Mesostigma *counterpart relative to the IR-containing genomes of *Nephroselmis *and representatives of the Streptophyta (Table 1). These results are congruent with the two best trees based on inversion medians that we recently inferred from streptophyte gene order data using *Mesostigma *as outgroup [8]. In these trees, the branch leading to *Chlorokybus *exhibits 9 or 10 inversions compared to the only 2 or 3 inversions observed for the branch leading to *Mesostigma*.

The intergenic regions of the *Chlorokybus *and *Mesostigma *genomes were surveyed for the presence of short repeated sequences (tandem repeats, palindromes and dispersed repeats). As estimated with RepeatMasker , short repeats represent only 1.4% and 0.7% of the intergenic regions in *Chlorokybus *and *Mesostigma *cpDNAs, respectively. While short repeats are also rare in other charophycean green algal genomes and in *Nephroselmis *cpDNA, they are much more abundant in the genomes of chlorophytes representing the Ulvophyceae, Trebouxiophyceae and Chlorophyceae (Table 2). Most of the *Chlorokybus *repeats consist of tandem repeats with repeat units ranging from 9 to 27 bp in size, whereas the repeats present in *Mesostigma *consist mainly of stem-loop structures of 26 to 55 bp.

### Phylogenetic inferences based on sequence data

**Figure 2:**
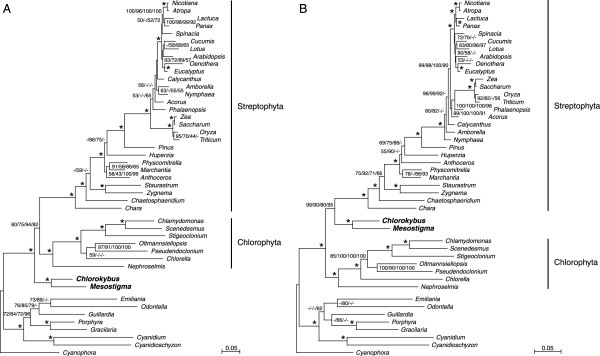
**Phylogenetic positions of *Chlorokybus *and *Mesostigma *within the Viridiplantae as inferred from sequences derived from the chloroplast genome**. (A) Best ML tree based on 45 proteins from 45 algal/plant taxa. (B) Best ML tree based on 45 genes from 45 algal/plant taxa. The eight non-green algae (bottom of the figure) were used as outgroup. The nodes that received 100% bootstrap support in ML, MP, ML-distance and LogDet-distance analyses are denoted by asterisks. For the other nodes, only the bootstrap values ≥50% are indicated; the values obtained in ML, MP, ML-distance, and LogDet-distance analyses are listed in this order from left to right. The detailed names of taxa are reported in the Methods. The 45 genes analyzed are as follows: *atpA*,*B*,*E*,*F*,*H*, *petB*,*D*,*G*, *psaA*,*B*,*C*,*J*, *psbA*,*B*,*C*,*D*,*E*,*F*,*H*,*I*,*J*,*K*,*N*,*T*,*Z*, *rpl2*,*14*,*16*,*20*,*36*, *rpoB*,*C1*,*C2*, *rps2*,*3*,*4*,*7*,*8*,*11*,*12*,*14*,*18*,*19*, *ycf3*,*4*.

To identify the phylogenetic positions of *Chlorokybus *and *Mesostigma *within the Viridiplantae, we first analyzed an amino acid data set containing a total of 8,657 sites (4,179 of which are phylogenetically informative) using maximum parsimony (MP), maximum likelihood (ML), ML distance and LogDet methods (Figure 2A). This data set was derived from 45 protein-coding genes common to the cpDNAs of 37 green algal/land plant taxa (Table 3) and eight non-green algae; the non-green algal sequences served as outgroup to root the trees. Unexpectedly, the best trees inferred with all four methods identified a clade uniting *Chlorokybus *and *Mesostigma*. This clade received 100% bootstrap support; however, its basal placement relative to the Streptophyta and Chlorophyta (topology T1; Figure 2A) was moderately supported, with 75% and 80% bootstrap values in MP and ML analyses, respectively. In the alternative T2 topology, the *Chlorokybus + Mesostigma *clade was identified as the first branch of the Streptophyta, whereas in the alternative T3 topology, it represented the most basal divergence of the Chlorophyta. Both T2 and T3 were recovered in ML and MP analyses, with T2 being better supported than T3 in ML analyses and the situation being reversed in MP analyses. Comparing these results with those reported by Lemieux *et al*. [13] indicates that the placement of *Mesostigma *at the base of the Chlorophyta and Streptophyta remained favoured upon broader taxon sampling but received weaker support. In contrast to the study of Lemieux *et al*. [13] in which both T2 and T3 proved to be significantly worse than T1 in confidence tests of tree selections, only the T3 topology was rejected at the 5% percent confidence level (T2, *P *= 0.135; T3, *P *= 0.031) in Approximately Unbiased (AU) tests.

The relationships observed for the other green algae and land plant taxa in the phylogeny shown in Figure 2A are congruent with recently published green plant phylogenies based on whole chloroplast genome sequences [8,32-37]. The clade formed by the two zygnematalean green algae (*Staurastrum *and *Zygnema*) is sister to all land plants and, as previously reported, this sister-relationship is weakly supported. The bryophytes (*Marchantia, Anthoceros formosae *and *Physcomitrella patens*) are sister to all other land plants, and again here, support for the monophyly of this group is weak to moderate. We find unambiguous support for the gymnosperm lineage (*Pinus thunbergii*), being sister to all angiosperms; however, the relationships among the members of the latter group are less resolved than in chloroplast phylogenies focusing on streptophytes and including a larger number of phylogenetically informative sites [8,32,34,35]. For example, our analysis fails to identify the monophyly of monocots although it provides strong support for the monophyly of eudicots (represented by ten taxa including *Nicotiana tabacum*, *Spinacia oleracea*, *Arabidopsis thaliana *and *Eucalyptus globulus*). The monocots *Acorus calamus *and *Phalaenopsis aphrodite *would be expected to cluster with the strongly supported clade uniting the grasses (*Zea mays*, *Saccharum officinarum*, *Oryza sativa *and *Triticum aestivum*). For the Chlorophyta, the basal divergence of the Prasinophyceae (*Nephroselmis*) relative to the Ulvophyceae (*Pseudendoclonium *and *Oltmannsiellopsis viridis*), Trebouxiophyceae (*Chlorella vulgaris*) and Chlorophyceae (*Scenedesmus, Chlamydomonas *and *Stigeoclonium helveticum*) is strongly supported, but the branching order of the latter three lineages is unclear. In agreement with chlorophyte phylogenies inferred from cpDNA-encoded proteins and genes [36], the Trebouxiophyceae are sister to the Ulvophyceae. In contrast, chloroplast phylogenies inferred from gene order [36] as well as mitochondrial phylogenies inferred from proteins or genes [38] revealed that the Ulvophyceae share a sister-relationship with the Chlorophyceae. Concerning the relationships among the other algae examined, our results agree with the chloroplast genome-based tree reported by Hagopian *et al*. [28] and with phylogenies inferred from smaller sets of chloroplast genes [39-41] and from nuclear-encoded plastid-targeted genes [42-44] in being consistent with the hypothesis that the chloroplasts of chromists (the chlorophyll *c*-containing cryptophytes, heterokonts and haptophytes) originated from a single secondary endosymbiotic event involving a red alga [45]. We found that *Guillardia, Odontella *and *Emiliania *form a moderately supported clade, which is sister to the strongly supported clade uniting the red algae *Porphyra *(Bangiales, Bangiophycidae) and *Gracilaria *(Florideophycidae). As expected, the two red algal taxa representing the Cyanidiales [*Cyanidium *and *Cyanidioschyzon *(Bangiophycidae)] robustly cluster in a separate clade.

The phylogenies that we inferred from a separate data set containing the chloroplast gene sequences (first and second codon positions) for all the proteins analyzed in Figure 2A proved to be more robust than the corresponding protein trees (Figure 2). The nucleotide data set comprised a total of 18,116 sites, 7,779 of which are phylogenetically informative. Better resolution of both internal and terminal nodes was observed for the portions of the gene trees corresponding to the Streptophyta and Chlorophyta. All four inference methods identified in 80% to 99% of the bootstrap replicates the strongly supported clade uniting *Chlorokybus *and *Mesostigma *as the deepest branch of the Streptophyta (T2 topology, Figure 2B). T1 was the only alternative topology observed in these analyses. This topology and the T3 topology were rejected at the 5% percent confidence level (T1, *P *= 0.028; T3, *P *= 7e-31) in AU tests.

**Figure 3:**
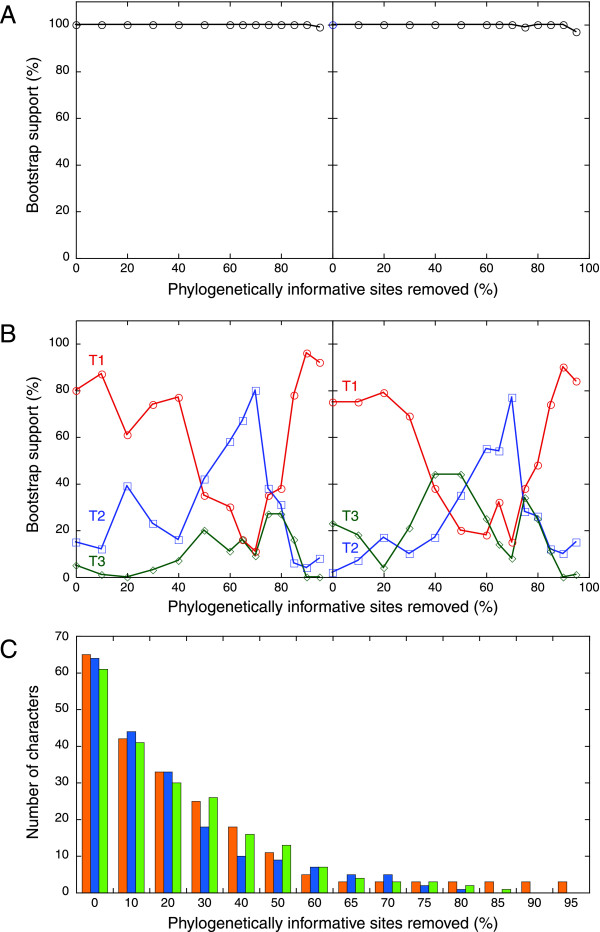
**Influence of removing increasing proportions of fast-evolving sites in the amino acid data set on the robustness of the nodes supporting the *Chlorokybus *+ *Mesostigma *clade and the three possible positions identified for this clade**. A series of data sets lacking 10% to 90% of the 4,179 phylogenetically informative sites present in the original data set analyzed in Figure 2A were subjected to phylogenetic analyses. (A) Fluctuations of bootstrap values for the node supporting the *Chlorokybus *+ *Mesostigma *clade in ML (left side) and MP (right side) analyses. (B) Fluctuations of bootstrap values for the nodes supporting the T1, T2 and T3 placement of the *Chlorokybus *+ *Mesostigma *clade in ML (left side) and MP (right side) analyses. The three topologies tested are colour-coded as follows: red, T1 topology, *i.e*. the placement of the clade before the divergence of the Streptophyta and Chlorophyta; blue, T2 topology, *i.e*. the placement of the clade as sister to the Streptophyta; and green, T3 topology, *i.e*. the placement of the clade as sister to the Chlorophyta. (C) Fluctuations in the number of characters supporting unambiguously each of the three topologies identified for the *Chlorokybus *+ *Mesostigma *clade. The topologies are colour-coded as in (B).

**Figure 4:**
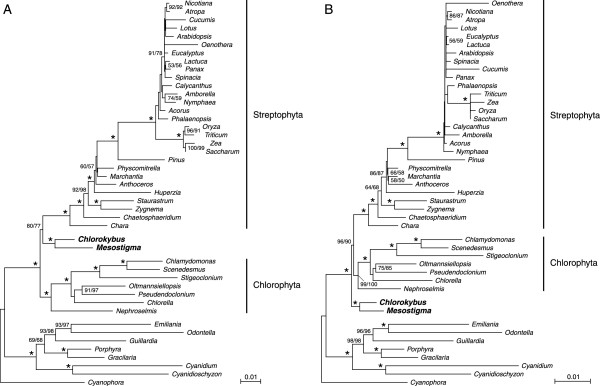
**Influence of removing substantial proportions of fast-evolving sites in the amino acid data set on the resolution of phylogenetic relationships**. (A) Best ML tree inferred from the data subset lacking 70% of the original information. (B) Best ML tree inferred from the data subset lacking 90% of the original information. The nodes that received 100% bootstrap support in ML and MP analyses are denoted by asterisks. For the other nodes, only the bootstrap values ≥ 50% are indicated; the values obtained in ML and MP analyses appear on the left and right, respectively.

Genome-based phylogenies are susceptible to artefacts in phylogeny reconstructions because they are inherently associated with limited taxon sampling [35,46,47]. Fast-evolving characters, in particular, are challenging for inference of such phylogenies because they are likely to have experienced many changes that mask the phylogenetic signal [48]. To examine whether these sites are a source of inconsistency in the protein phylogenies shown in Figure 2A, we analyzed subsets of the original data in which increasing proportions of the fastest-evolving sites were removed. Figure 3 shows the effect of excluding 10% to 90% of the phylogenetically informative sites on the robustness of the *Chlorokybus *+ *Mesostigma *clade and on the robustness of the T1, T2 and T3 topologies in both ML and MP analyses. Whatever the subset of data examined, *Chlorokybus *and *Mesostigma *remained strongly affiliated in the same clade (Figure 3A). After excluding up to 30% or 40% of the phylogenetically informative sites, the T1 topology was still moderately supported, with bootstrap values varying from 61% to 87% (Figure 3B). However, consistent with the idea that the fastest-evolving sites are a source of phylogenetic inconsistency, removal of 50% to 80% of the phylogenetically informative sites resulted in a substantial decline in the robustness of T1 and a concomitant increase in the level of support observed for T2 and/or T3, with T2 receiving a maximal support level of about 80% upon removal of 70% of the informative sites (Figure 3B). Intriguingly, when fastest-evolving sites were further removed, the T1 topology became more robust and received maximal bootstrap support levels of 96% and 90% in ML and MP analyses, respectively (Figure 3B). Despite extensive loss of the original information, many nodes in the best ML tree shown in Figure 2A remained strongly supported (Figure 4).

In the above analyses focusing on the most reliable slow-evolving characters present in the original amino acid data set, we have also followed the evolution of the phylogenetic signal by tracing the characters supporting unambiguously the T1, T2 or T3 topology (Figure 3C). A comparable number of characters support unambiguously each of these topologies in the original data set and most subsets (up to 80% site removal). Importantly, the vast majority of the approximately 65 characters supporting each topology in the original data set fall within the fastest-evolving sites. After removing 50% of the phylogenetic information, less than 15 unambiguously supporting characters were identified for each topology, and exclusion of more than 85% of the information led to complete loss of the characters providing unambiguous support for T2 and T3, thus explaining the prevalent recovery of T1 in analyses of the corresponding data subsets (Figure 3B).

**Figure 5:**
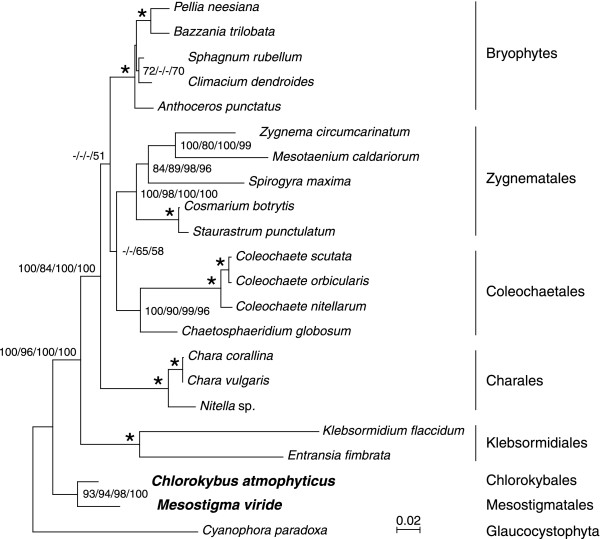
**Phylogenetic positions of *Chlorokybus *and *Mesostigma *as inferred from chloroplast small and large subunit rRNA genes**. The *Chlorokybus *rRNA gene sequences were added to a data set previously analyzed by Turmel *et al*. [16] and phylogenetic analyses of the resulting data set (4,072 sites) were carried out essentially as described in the Methods, except that the TrN+Γ+I model was used. The best ML tree is shown. The nodes that received 100% bootstrap support in ML, MP, ML-distance and LogDet-distance analyses are denoted by asterisks. For the other nodes, only the bootstrap values ≥50% are indicated; the values obtained in ML, MP, ML-distance, and LogDet-distance analyses are listed in this order from left to right.

Given that a representative of the Klebsormidiales was not included in the above analyses, incomplete taxon sampling of the charophycean green algae might have led to the artefactual clustering of *Chlorokybus *with *Mesostigma*. To investigate the relationship between *Chlorokybus *and the Klebsormidiales, we inferred phylogenies from the chloroplast small and large subunit rRNA genes of *Mesostigma, Chlorokybus, Klebsormidium, Entransia*, and 17 other streptophytes (Figure 5). Support for the specific affinity between *Mesostigma *and *Chlorokybus *remained very robust in these analyses, and consistent with the four-gene tree of Karol *et al*. [4] and our previous phylogenetic study based on chloroplast rRNA genes [16], *Klebsormidium *and *Entransia *formed a lineage that is sister to the clade uniting the Charales, Coleochaetales, Zynematales and land plants.

### Phylogenetic inferences based on structural data

**Figure 6:**
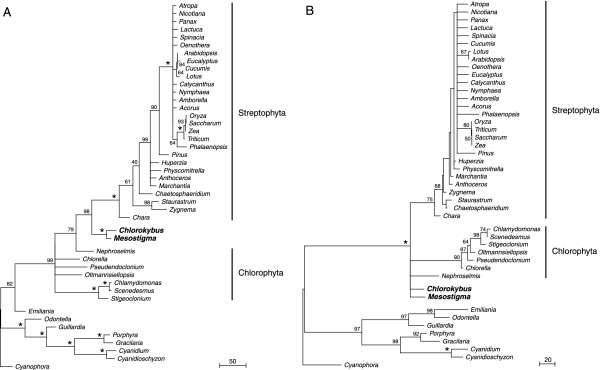
**Phylogenetic positions of *Chlorokybus *and *Mesostigma *within the Viridiplantae as inferred from structural features of the chloroplast genome**. (A) MP analysis based on gene content. The strict consensus of 12 equally parsimonious trees is shown. (B) MP analysis based on gene order. The strict consensus of 56 equally parsimonious trees is shown. The nodes that received 100% bootstrap support are denoted by asterisks. For the other nodes, only the bootstrap values ≥50% are indicated.

To gain independent information concerning the phylogenetic position of the *Mesostigma *+ *Chlorokybus *clade, we examined structural features of the chloroplast genome (gene order and gene content) from the same taxa used in our phylogenetic analyses of protein and gene sequences. MP analysis of the gene order data alone (525 characters) confirmed the close affinity of *Mesostigma *to *Chlorokybus *(29 characters are specifically shared by these algae) and showed that the clade uniting these two algae represents a basal divergence of the Streptophyta (Figure 6A). Although relationships were not as well resolved as in the phylogenies inferred from sequence data (see Figure 2), they were found to be generally congruent with these phylogenies. The failure to identify the chlorophytes as a monophyletic group is probably related to the dramatic differences in gene order observed in this group [23,36,49-51]. Likewise, the inclusion of *Emiliania *within the clade containing all chlorophytes and streptophytes probably stems from the considerable gene order divergence displayed by this haptophyte compared to the two other algae carrying secondary chloroplasts and the red algae [31].

MP analysis of gene content yielded a phylogeny more poorly resolved than that inferred from gene order (Figure 6B). Although this analysis failed to identify the *Chlorokybus *+ *Mesostigma *clade and most of the streptophyte clades observed in the best trees inferred from sequence data, it clustered the chlorophytes belonging to the Trebouxiophyceae, Ulvophyceae and Chlorophyceae together in a highly supported clade and identified a sister-relationship for the Chlorophyceae and Ulvophyceae. Similarly, the relationships observed for the chloroplasts of the red algae and secondary chloroplasts were well resolved, revealing a clade uniting the red algal chloroplasts and a sister clade clustering the secondary chloroplasts.

**Figure 7:**
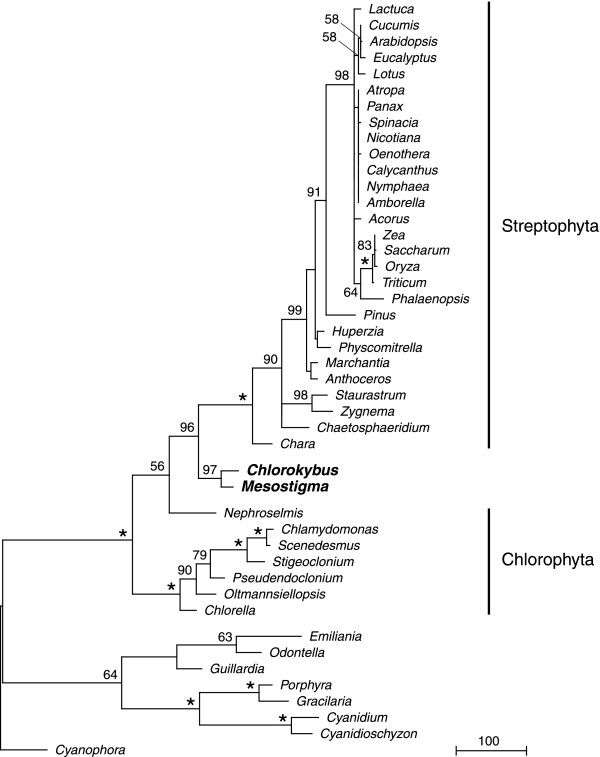
**MP analysis based on combined gene content and gene order data derived from the chloroplast genomes**. The figure shows the strict consensus of 28 equally most parsimonious trees. The nodes that received 100% bootstrap support are denoted by asterisks. For the other nodes, only the bootstrap values ≥ 50% are indicated.

MP analysis of combined gene order and gene content data proved to have a better resolving power than the analyses based on the individual data alone, even though bootstrap support for some nodes were not significantly higher (Figure 7). The *Chlorokybus *+ *Mesostigma *clade was identified as the most basal divergence of the Streptophyta and the monophyly of all chlorophytes, except *Nephroselmis*, was observed. With regards to the red algal lineage, the red algal chloroplasts formed a strongly supported monophyletic group, whereas the clade clustering the secondary chloroplasts received low bootstrap support.

## Discussion

### *Mesostigma *and *Chlorokybus *are sister taxa

Our finding that the *Chlorokybus *chloroplast genome shows remarkable similarity in gene content and gene order with its *Mesostigma *homologue is entirely congruent with our phylogenetic inferences based on whole chloroplast genome data in indicating a close alliance between *Chlorokybus *and *Mesostigma*. In trees inferred from all data sets examined in this study, except the gene content data set, these two green algae form a strongly supported clade that either branches basally within the Streptophyta or before the split of the Streptophyta and Chlorophyta. The evidence for a sister relationship between *Mesostigma *and *Chlorokybus *is particularly compelling considering that analyses of gene order and sequence data independently support this relationship.

Because *Mesostigma *and *Chlorokybus *differ in cellular organization and habitat, the sister relationship shared by these two green algae indicates that important changes occurred at these levels in the lineage leading to *Chlorokybus*. More specifically, colonies made up of sarcinoid, cubical packets of non-flagellated vegetative cells and occurring in subaerial habitats (mainly on rocky substrata) evolved from unicellular, scaly biflagellates living exclusively in aquatic habitats. The opposite scenario in which *Mesostigma *took origin from a '*Chlorokybus*-like' zoospore evolving into a free-living flagellate can be discarded because it is less parsimonious for the following three reasons. First, all early-diverging lineages of the Chlorophyta comprise primarily flagellates; second, given that *Mesostigma *has two multi-layered structures in its flagellar apparatus instead of a single one as in *Chlorokybus *and is the only streptophyte featuring an eyespot, the transformation of *Chlorokybus*-like zoospores into *Mesostigma*-like cells would require the gain of an eyespot and of an additional multi-layered structure; and third, recent evidence suggests that sarcinoid chlorophytes arose from unicells on multiple occasions [52]. Considering that *Mesostigma *reflects a more ancestral condition than *Chlorokybus*, the sarcinoid cellular organization of *Chlorokybus *can no longer be viewed as an intermediate step in the pathway leading to multicellularity [7]; according to the evolutionary scenario reported here, the filamentous cellular organization displayed by streptophyte green algae belonging to the Klebsormidiales originated independently of the sarcinoid condition from a biflagellate ancestor.

Since the discovery of *Chlorokybus *by Geitler in 1942 [53], a range of divergent views have been expressed concerning its classification. This rare green alga, which Geitler observed in only two locations in Austria, had been considered to belong to various orders of the Chlorophyceae until Rogers *et al*. [54] placed it in the newly erected charophycean order Chlorokybales on the basis of the ultrastructure of the flagellar apparatus. Vegetative cells of *Chlorokybus *can be induced to produce flagellated cells, also called zoospores. As observed for the flagellated cells of all charophyceans and *Mesostigma*, Rogers *et al*. [54] found that the body and flagella of the *Chlorokybus *zoospores are covered with small square scales and that the laterally inserted flagella are attached internally to a multilayered structure. More recently, based on his studies of the mitotic and cytokinetic patterns of vegetative cells, Lokhorst *et al*. [55] proposed to remove *Chlorokybus *from the Chlorokybales and merge it in the Klebsormidiales.

In the present study, we could not investigate the relationship of *Chlorokybus *with members of the Klebsormidiales; however, it is unlikely that the inclusion of klebsormidialean green algae in our phylogenies would have abolished the specific affinity we uncovered between *Mesostigma *and *Chlorokybus*. Indeed, these two algae remained robustly clustered when we inferred phylogenies from the chloroplast small and large subunit rRNA genes of *Mesostigma, Chlorokybus, Klebsormidium, Entransia*, and 17 other streptophytes (Figure 5). Moreover, the chlorokybalean and klebsormidialean lineages clearly represent separate branches in the four-gene tree of Karol *et al*. [4].

### The *Mesostigma *+ *Chlorokybus *clade occupies the deepest branch of the Streptophyta

The phylogenies reported here shed new light into the controversy regarding the position of *Mesostigma *within the Viridiplantae. The strong clustering of *Mesostigma *with *Chlorokybus*, an alga that is without any doubt a streptophyte with regards to its cellular organization, provides unambiguous evidence that *Mesostigma *belongs to the Streptophyta. Solid evidence for the positioning of *Mesostigma *within the Streptophyta also comes from the observation that trees inferred from chloroplast gene sequences and gene order data robustly resolve the *Mesostigma *+ *Chlorokybus *clade as the deepest branch of the Streptophyta (Figure 2B). We are confident that these lines of evidence based on chloroplast genome data reflect the true organismal relationship of *Mesostigma *with streptophytes because they are consistent with phylogenetic and EST data derived from separate cellular compartments. Like the gene phylogenies reported here, a number of phylogenetic analyses support the affiliation of *Mesostigma *with streptophytes [4,10-12], and in agreement with this relationship, recent analyses of EST data from *Mesostigma *revealed nuclear genes that appear to be specific to streptophytes [18-20].

It appears that the placement of the *Mesostigma *+ *Chlorokybus *clade before the divergence of the Streptophyta and Chlorophyta in our analyses of the amino acid data set corresponding to the gene data set is the result of phylogenetic inconsistencies. Although this topology was recovered with moderate bootstrap support by all methods of phylogenetic inference (Figure 2A), our analyses of data subsets progressively enriched in slow-evolving characters suggest that it is incorrect (Figure 3). When about 70% of the fastest-evolving sites in the original data set were removed, the placement of the *Mesostigma *+ *Chlorokybus *clade within the Streptophyta was favoured with moderate support; however, further exclusion of phylogenetically informative sites led to the re-emergence of the topology positioning this clade before the divergence of the Streptophyta and Chlorophyta. In light of these results and of the overwhelming evidence supporting the affiliation of the *Mesostigma *+ *Chlorokybus *clade with the Streptophyta (see above), we conclude that the phylogenetic signal in the original amino acid data set was masked by conflicting (non-phylogenetic) signals.

### Current issues in chloroplast phylogenomic studies

Our study provides another example of the importance of taxon sampling in phylogenomic studies. The use of complete chloroplast genome data in phylogenetic analyses of green algae and land plants has been implemented as a powerful alternative to the traditional approach based on a few genes from many taxa. This whole-genome approach, however, has been strongly criticized because it can yield statistically well-supported trees that do not reflect true organismal relationships as a result of sparse taxon sampling [47]. The debate on the taxon-dense versus character-rich approaches has focused on three high profile cases of chloroplast phylogenomic studies: the earliest angiosperms [14,34,56-59], the deepest branch of the land plants [33,60,61] and the most basal divergence of the Viridiplantae/Streptophyta [4,10,11,13,16,17]. In all three cases, the tree topologies inferred from chloroplast genome data have now been shown to be sensitive to taxon sampling and the addition of taxa has been instrumental in resolving the conflicts between the character-rich and taxon-dense data sets. In the case of the earliest angiosperms, the addition of basal monocots [35,62] and magnolids [63] has strengthened the notion that either *Amborella *or a clade containing *Amborella *and the Nymphaeales is sister to all other angiosperms. With regards to the deepest divergence of the land plants, addition of a single lineage, the lycophytes, had a dramatic effect on the resolution of the liverworts, mosses, hornworts and vascular plants, providing support for the liverworts being sister to all other land plants and the hornworts being sister to vascular plants [8,37,61]. Finally, as reported in this study, analyses of chloroplast genome data sets supplemented with several streptophyte and chlorophyte taxa no longer support *Mesostigma *as sister to all other green algae and land plants but rather favour the notion that this alga occupies the earliest branch of the Streptophyta.

Aside from sparse taxon sampling, a number of other factors can compromise the performance of phylogenetic reconstruction methods in chloroplast phylogenomic studies [14,57]. These include misspecifications of the evolutionary models employed, compositional heterogeneity of the data sets and evolutionary rate heterogeneity among different characters and lineages. In this context, it is worth discussing the utility of the amino acid versus nucleotide data sets in phylogenetic analyses of green algae and land plants. Amino acid sequences have frequently been used in the past to infer deep phylogenies because they avoid problems with saturation of silent substitutions and differential G+C content. However, our study has clearly shown that the nucleotide data set greatly outperformed the deduced amino acid data set in its ability to identify the true phylogenetic position of the *Chlorokybus *+ *Mesostigma *clade. Nucleotide data were also found to be superior to amino acid data in studies aimed at identifying the deepest divergence of the land plants [8,61]. These observations suggest that the nucleotide data sets are not as saturated and biased in base composition as the divergence time of the streptophyte lineages under study would predict. The exact cause of our failure to recover the true position of the *Chlorokybus *+ *Mesostigma *clade with the amino acid data set remains unclear. One of the possible explanations is that the empirical model of amino acid replacement (cpREV) used to reconstruct the evolution of chloroplast proteomes in ML analyses is not optimal for green algae and land plants. This model of amino acid substitution was derived from 45 proteins encoded in the chloroplast genomes of *Cyanophora*, a diatom, a red alga, a euglenid and five land plants [64]. A more realistic model of amino acid substitution derived from a broad sampling of the Viridiplantae could help to resolve more accurately the deep branches of this phylum.

Considering the numerous potential problems associated with tree reconstructions in phylogenomic studies, the phylogenies inferred in these studies need to be validated with independent data sets before concluding that they reflect true organismal relationships. Candidate sources of independent phylogenetic data are diverse and include sequence data from other genomes as well as structural genomic and morphological data.

### The shared ancestry of *Mesostigma *and *Chlorokybus *alters our view of chloroplast genome evolution in the Viridiplantae

The shared streptophyte ancestry of *Mesostigma *and *Chlorokybus *reveals that the chloroplast genome of the common ancestor of all streptophytes was richer in genes than previously thought. We infer that this ancestral genome harboured a minimum of 144 genes, 17 of which are not found in the Chlorophyta [bioY*, ndhJ, odpB, rbcR*, rpl21, rpl22, rpl33, rps15, rps16, ssrA*, ycf27*, ycf61*, ycf65*, ycf66, trnA(ggc)*, trnP(ggg), and *trnV*(gac), where asterisks denote the genes present only in *Mesostigma *and/or *Chlorokybus*]. The chloroplast genome sequences currently available for chlorophytes suggest that the gene repertoire of the common ancestor of these algae was smaller and included 131 genes, only four of which are not present in the Streptophyta [*rne, rnpB, ycf47*, and *trnR*(ccu)].

Changes in gene content, gene order, and in the size of intergenic regions mainly account for the differences between the *Mesostigma *and *Chlorokybus *chloroplast genomes. Seven gene losses [*accD, rbcR, ycf27*, and *trnR*(ccg) in *Mesostigma*, and *bioY, ssrA*, and *ycf81 *in *Chlorokybus*] and a minimum of 14 inversions distinguish these two genomes. Compared to their *Mesostigma *counterparts, the *Chlorokybus *intergenic regions accumulated a larger proportion of short dispersed repeats and grew significantly in size, resulting in a gene density comparable to that observed in the loosely packed genomes of chlorophytes belonging to the Trebouxiophyceae, Ulvophyceae, and Chlorophyceae. The higher abundance of short dispersed repeats might have provided more opportunities for recombination between these elements and thus may explain the higher rate of gene rearrangements observed in the *Chlorokybus *lineage [8].

## Conclusion

In disclosing a sister relationship between the biflagellate *Mesostigma *and the sarcinoid *Chlorokybus*, our study alters substantially our concepts regarding the evolution of streptophyte algae and closes the long-standing debate on the phylogenetic position of *Mesostigma *within the Viridiplantae. The weight of evidence supporting the notion that streptophyte algae took their origin from a unicellular freshwater flagellate like *Mesostigma *has now become overwhelming and in the future, this hypothesis should gain further support from phylogenetic analysis of EST data. In predicting a richer chloroplast gene repertoire than previously inferred for the common ancestor of all streptophytes, our study has also a significant impact on chloroplast genome evolution in the Viridiplantae. The chloroplast gene repertoires of *Mesostigma *and *Chlorokybus *are the largest known among green algae and include several genes that are present in non-green algae but are absent from all other green algal cpDNAs investigated thus far.

## Methods

### DNA cloning, sequencing and sequence analysis

*Chlorokybus atmophyticus *was obtained from the Sammlung von Algenkulturen Göttingen (SAG 48.80) and grown in medium C [65] under 12 h light/dark cycles. A random clone library was prepared from a fraction containing both cpDNA and mitochondrial DNA [66]. DNA templates were obtained with the QIAprep 96 Miniprep kit (Qiagen Inc., Mississauga, Canada) and sequenced as described previously [22]. Sequences were edited and assembled using SEQUENCHER 4.1.1 (Gene Codes Corporation, Ann Arbor, MI, USA). The fully annotated chloroplast genome sequence has been deposited in [GenBank:DQ422812].

Genes and ORFs were identified as described previously [36]. Repeated sequences were identified with REPuter 2.74 [67] using the -f (forward), -p (palindromic), and -allmax options at minimum lengths of 30 bp and were classified with REPEATFINDER [68]. Number of copies of each repeat unit was determined with FINDPATTERNS of the Wisconsin package version 10.3 (Accelrys, San Diego, CA, USA). Stem-loop structures and direct repeats were identified using PALINDROME and ETANDEM in EMBOSS 2.9.0 [69], respectively. Genomic regions containing non-overlapping repeated elements were identified with RepeatMasker  running under the WU-BLAST 2.0  search engine.

### Analysis of genome rearrangements

The GRIMM web server [70] was used to infer the number of gene permutations by inversions in a comparison of *Chlorokybus *and *Mesostigma *cpDNAs as well as in pairwise comparisons involving either *Chlorokybus *or *Mesostigma *cpDNA with selected IR-containing genomes. For these analyses, genes within one of the two copies of the IR were excluded from the data set, and the SSC and LSC + IR regions were considered as two separate chromosomes. The SSC and LSC regions were assumed to be independent from one another because the conserved gene partitioning pattern displayed by the examined genomes is not consistent with the occurrence of inversions spanning the SSC and LSC regions.

### Phylogenetic inferences from sequence data

GenBank files were retrieved for the 37 green algal/land plant chloroplast genomes listed in Table 3 and for the following eight non-green algal chloroplast genomes: *Cyanidioschyzon merolae *(GenBank:NC_004799), *Cyanidium caldarium *(GenBank:NC_001840), *Cyanophora paradoxa *(GenBank:NC_001675), *Emiliania huxleyi *(GenBank:NC_007288), *Gracilaria tenuistipitata *(GenBank:NC_006137), *Guillardia theta *(GenBank:NC_000926), *Odontella sinensis *(GenBank:NC_001713), *Porphyra purpurea *(GenBank:NC_000925). All GenBank files were revised to ascertain that all genes in each genome are identified and annotated using the same gene designations. The chloroplast genome sequences of the euglenid *Euglena gracilis *(GenBank:NC_001603) and the chlorarachniophyte *Bigelowiella natans *(GenBank:NC_008408) were not sampled in this study because they produce long branches in phylogenetic analyses that could lead to wrong topologies [15]. The chloroplasts of these taxa were secondarily acquired from green algae through independent endosymbiotic events.

A data set of 45 concatenated protein sequences was derived as described previously [66] from all protein-coding genes common to the above chloroplast genomes, except *rbcL *(a gene existing as two distinct forms in red and green algal lineages and possibly implicated in an horizontal transfer event [71]). Phylogenetic analyses of this data set were carried out using ML, MP, ML-distance and LogDet-distance methods. ML trees were computed with PHYML 2.4.5 [72] under the cpREV45+Γ+I model of amino acid substitutions [64] and bootstrap support for each node was calculated using 100 replicates. MP trees and ML-distance trees were inferred using PROTPARS and NEIGHBOR, respectively, in PHYLIP 3.65 [73]. The ML distances were computed with PUZZLEBOOT 1.03 and TREE-PUZZLE 5.2 [74] under the cpREV45+Γ+I model. Robustness of MP and distance trees was assessed by bootstrap percentages after 100 replications. LogDet-distance trees were computed using PAUP 4.0b10 [75] with the neighbour-joining search setting. The LogDet-distances were calculated with LDDist [76], and the proportion of invariant sites was estimated using the capture-recapture method of Steel *et al*. [77]. Confidence of branch points was estimated by 1,000 bootstrap replications.

A data set containing the gene sequences (first two codon positions only) coding for the 45 proteins represented in the amino acid data set was also analyzed using various methods of phylogenetic inference. This nucleotide data set was prepared as described previously [8]. ML trees were inferred using PHYML 2.4.5, whereas MP and ML-distance trees were inferred using PAUP 4.0b10. In MP analysis, trees were searched with the full heuristic option and optimization was performed by branch-swapping using tree bisection and reconnection; in ML-distance analysis, trees were searched with the neighbour-joining search setting. ML and ML-distance trees were constructed under the GTR+Γ+I model using the parameters estimated by PHYML. Confidence of branch points was estimated by 100 bootstrap replications in ML and MP analyses and 1,000 bootstrap replications in ML-distance analysis. LogDet-distance trees were computed using PAUP 4.0b10 with the neighbour-joining search setting. The LogDet-distances were calculated with LDDist, and the proportion of invariant sites was estimated using the capture-recapture method of Steel *et al*. [77]. Confidence of branch points was estimated by 1,000 bootstrap replications.

AU tests [78] were performed with CONSEL 0.1i [79] on the amino acid and nucleotide data sets to compare the three alternative positions of the *Chlorokybus *+ *Mesostigma *clade. Test trees were constructed as follows: ML phylogenies excluding *Chlorokybus *and *Mesostigma *were optimized using PHYML and the abovementioned evolutionary models, and then the *Chlorokybus *+ *Mesostigma *clade was added to positions corresponding to the T1, T2 and T3 topologies. Site-wise log-likelihoods for each test tree were computed with TREE-PUZZLE 5.2 [74] using the -wsl options.

The influence of removing increasing proportions of fast-evolving sites in the amino acid data set was investigated as follows. Substitution rates among sites in the data set were estimated with CODEML for the best trees inferred by ML and MP, and these rates were averaged for each site. Phylogenetically informative sites were incrementally removed by order of decreasing rate of evolution to generate 13 subsets of data. ML and MP analyses of these subsets were performed as described above for the original amino acid data set. The number of phylogenetically informative sites supporting unambiguously the placement of the clade uniting *Chlorokybus *and *Mesostigma *at each of the three possible positions in the global phylogeny was identified using MacClade 4.08 [80].

### Phylogenetic inferences from structural genomic data

A data set of gene content was prepared from the chloroplast genomes of the 45 taxa listed above by coding the presence of a gene, the presence of a pseudogene, and the absence of a gene as Dollo characters with values of 2, 1 and 0, respectively. Gene order in each of these chloroplast genomes was converted to all possible pairs of signed genes (*i.e*., taking into account gene polarity) and a gene order data set was obtained by coding as binary characters the presence/absence of signed gene pairs in two or more genomes. The gene content and gene order data sets were merged together to produce a data set of combined structural data. Each of the three data sets was subjected to MP analysis under the Dollo principle (*i.e.*, assuming that characters can be lost independently in several evolutionary lineages but cannot be regained [81]) using PAUP 4.0b10. Confidence of branch points was estimated by 100 bootstrap replications. MacClade 4.08 was used to generate the matrices of gene content and gene order data, to trace the encoded characters on tree topologies, and to calculate tree lengths.

## Authors' contributions

CL and MT conceived and designed the study, and wrote the manuscript. CL performed most of the sequence analyses, and generated the figures. MT also contributed to the analysis and interpretation of the data. CO carried out the sequencing of the *Chlorokybus *chloroplast genome and identified the repeated sequence elements in this genome. All authors read and approved the final manuscript.

## Figures and Tables

**Table 1:** Conservation of gene order in green algal and land plant cpDNAs relative to their *Chlorokybus *and *Mesostigma *homologues

**Compared genomes**	**No. of conserved gene clusters ^a^**	**No. of genes in clusters/no. of shared genes ^b^**	**Similarity index ^c^**	**No. of inversions ^d^**
***Chlorokybus***				
*Mesostigma*	15	126/134	6.3	12
*Chara*	18	93/126	4.1	42
*Chaetosphaeridium*	21	90/124	3.5	46
*Marchantia*	19	87/120	3.8	42
*Nephroselmis*	21	81/123	3.1	55
				
***Mesostigma***				
*Chlorokybus*	15	126/134	6.3	12
*Chara*	21	101/124	3.9	36
*Chaetosphaeridium*	23	96/122	3.4	42
*Marchantia*	22	95/118	3.7	39
*Nephroselmis*	24	89/122	3.0	50

^a ^A conserved cluster was defined as a suite of genes/gene pieces with the same order and polarities in the pair of compared genomes.

^b ^The ratio of these two values represents the fraction of shared genes/gene pieces found in conserved clusters.

^c ^This value was obtained by dividing the percentage of shared genes/gene pieces found in conserved clusters by the number of conserved clusters.

^d ^The GRIMM web server [70] was used to infer the numbers of inversions required to transform the gene order displayed by *Chlorokybus *or *Mesostigma *cpDNA into those of the compared genomes. In this analysis, the order of the 109 genes/gene pieces shared by the six compared genomes was examined.

**Table 2:** Abundance of repeats in *Chlorokybus *and other green algal cpDNAs

	**Non-overlapping repeats ^a^**		
	
**cpDNA**	**Total size (bp)**	**Fraction of genome (%)**	**Fraction of intergenic regions (%) ^b^**
**Streptophytes**			
*Mesostigma*	217	0.2	0.7
*Chlorokybus*	873	0.6	1.4
*Chara*	3,008	1.6	4.2
*Chaetosphaeridium*	972	0.7	3.2
*Zygnema*	2,226	1.3	3.2
*Staurastrum*	501	0.3	0.8
**Chlorophytes**			
*Nephroselmis*	1,061	0.5	1.1
*Chlorella*	11,743	7.8	14.9
*Oltmannsiellopsis*	18,033	11.9	29.9
*Pseudendoclonium*	10,621	5.4	12.9
*Stigeoclonium*	39,941	17.8	38.2
*Scenedesmus*	4,817	3.0	8.3
*Chlamydomonas*	34,244	16.8	34.2

^a ^Non-overlapping repeat elements were mapped on each genome with RepeatMasker using the repeats ≥ 30 bp identified with REPuter [67] as input sequences.

^b ^Unique ORFs were considered to be non-coding sequences, *i.e. *components of intergenic regions.

**Table 3:** Green algal and land plant chloroplast genomes examined in this study

**Green alga/land plant**	**Size (bp)**	**IR size (bp)**	**Genes (no.) ^a^**	**Introns (no.) ^b^**	**GenBank accession no.**
**Charophyceae**					
*Chara vulgaris*	184,933	10,919	127	2/16	GenBank:NC_008097
*Chaetosphaeridium globosum*	131,183	12,431	125	1/17	GenBank:NC_004115
*Chlorokybus atmophyticus*	152,254	7,640	138	1/0	GenBank:DQ422812
*Mesostigma viride*	118,360	6,057	137	0	GenBank:NC_002186
*Staurastrum punctulatum*	157,089	-	121	1/7	GenBank:NC_008116
*Zygnema circumcarinatum*	165,372	-	125	1/12	GenBank:NC_008117
					
**Land Plants**					
*Acorus calamus*	153,821	25,697	110	1/20	GenBank:NC_007407
*Amborella trichopoda*	162,686	26,651	111	1/20	GenBank:NC_005086
*Anthoceros formosae*	161,162	15,744	120	2/20	GenBank:NC_004543
*Arabidopsis thaliana*	154,478	26,264	110	1/20	GenBank:NC_000932
*Atropa belladonna*	156,687	17,031	111	1/20	GenBank:NC_004561
*Calycanthus floridus*	153,337	23,296	111	1/20	GenBank:NC_004993
*Cucumis sativus*	155,293	24,753	111	1/20	GenBank:NC_007144
*Eucalyptus globulus*	160,286	26393	109	1/20	GenBank:NC_008115
*Huperzia lucidula*	154,373	15,314	119	1/20	GenBank:NC_006861
*Lactuca sativa*	152,765	25,033	111	1/20	GenBank:NC_007578
*Lotus japonicus*	150,519	25,156	109	1/20	GenBank:NC_002694
*Marchantia polymorpha*	121,024	10,058	120	1/19	GenBank:NC_001319
*Nicotiana tabacum*	155,939	25,341	110	1/20	GenBank:NC_001879
*Nymphaea alba*	159,930	25,177	111	1/20	GenBank:NC_006050
*Oenothera elata*	163,935	27,807	109	1/18	GenBank:NC_002693
*Oryza sativa*	134,525	20,799	108	1/17	GenBank:NC_001320
*Panax ginseng*	156,318	26,071	111	1/20	GenBank:NC_006290
*Phalaenopsis aphrodite*	148,964	23,787	99	1/18	GenBank:NC_007499
*Physcomitrella patens*	122,890	9,589	116	1/20	GenBank:NC_005087
*Pinus thunbergii*	119,707	-	106	1/15	GenBank:NC_001631
*Saccharum officinarum*	141,182	22795	108	1/17	GenBank:NC_006084
*Spinacia oleracea*	150,725	25,073	110	1/19	GenBank:NC_002202
*Triticum aestivum*	134,545	20,703	108	1/17	GenBank:NC_002762
*Zea mays*	140,384	22,748	108	1/17	GenBank:NC_001666
					
**Prasinophyceae**					
*Nephroselmis olivacea*	200,799	46,137	128	0	GenBank:NC_000927
					
**Trebouxiophyceae**					
*Chlorella vulgaris*	150,613	-	112	3/0	GenBank:NC_001865
					
**Ulvophyceae**					
*Oltmannsiellopsis viridis*	151,933	18,510	104	5/0	GenBank:NC_008099
*Pseudendoclonium akinetum*	195,867	6,039	105	27/0	GenBank:NC_008114
					
**Chlorophyceae**					
*Chlamydomonas reinhardtii*	203,827	22,211	94	5/2	GenBank:NC_005353
*Scenedesmus obliquus*	161,452	12,022	96	7/2	GenBank:NC_008101
*Stigeoclonium helveticum*	223,902	-	97	16/5	GenBank:DQ630521

^a ^Genes present in the IR were counted only once. Pseudogenes, unique ORFs and intron ORFs were not taken into account.

^b ^Numbers of group I and group II introns are given before and after the slash, respectively.
